# 2542: Incidence of T3a up-staging and survival after partial nephrectomy: Size-stratified rates and implications for prognosis

**DOI:** 10.1017/cts.2017.293

**Published:** 2018-05-10

**Authors:** Arnav Srivastava, Hiten Patel, Max Kates, Zeyad Schwen, Gregory Joice, Alice Semerjian, Michael Gorin, Phillip Pierorazio, Mohammad E. Allaf

**Affiliations:** Johns Hopkins University School of Medicine, Baltimore, MD, USA

## Abstract

OBJECTIVES/SPECIFIC AIMS: Due to increased experience and favorable outcomes, the use of partial nephrectomy (PN) to treat renal cell carcinoma has grown in the past decade, with expansion to larger tumors. Performing PN for larger tumors could potentially increase the number of patients up-staged to pT3a after surgery, who may have instead been treated with radical nephrectomy (RN), if known preoperatively. We aimed to estimate the proportion of patients up-staged to T3a disease after PN stratified by size. We also compared size-stratified survival outcomes of up-staged patients to those with T1a, T1b, or T2 kidney cancer. METHODS/STUDY POPULATION: From 1998 to 2013, patients undergoing PN or RN were identified from Surveillance Epidemiology and End Results registries. The proportion of patients receiving PN found to have pT3a disease was quantified by size. Cox proportional hazards models compared cancer-specific (CSS) and overall survival (OS) for PN patients with pT1a, pT1b, and pT2 disease with appropriately size-stratified pT3a patients. Also, PN patients with pT3a disease were compared to size-stratified RN patients with pT3a disease. Comparisons by size were performed within pT3a patients receiving PN. RESULTS/ANTICIPATED RESULTS: From a total of 28,854 patients undergoing PN, the estimated proportion up-staged to pT3a increased along with increasing tumor size: 4.2% for T1a, 9.5% for T1b, and 19.5% for T2. Among patients receiving PN, adjusted survival analysis demonstrated worse CSS for up-staged pT3a patients Versus appropriately stratified pT1a (CSS: HR=1.87, *p*=0.02), pT1b (CSS: HR=1.91, *p*=0.01), and pT2 (CSS: HR=2.33, *p*=0.01) patients. However, when assessing OS, only the size-stratified comparison of up-staged pT3a Versus pT1a disease demonstrated worse OS for the up-staged cohort (OS: HR=1.25, *p*=0.04). Comparing PN and RN for pT3a disease, size-adjusted analysis revealed no statistical difference in CSS or OS. Lastly, among patients undergoing PN with pT3a disease, patients with larger tumors, measuring 4–7 cm (CSS: HR=2.83, *p*<0.01; OS: HR=1.44, *p*=0.04) or 7–16 cm (CSS: HR=8.22, *p*<0.01; OS: HR=2.64, *p*<0.01), experienced worse survival than those with smaller pT3a tumors, <4 cm. DISCUSSION/SIGNIFICANCE OF IMPACT: A greater proportion of patients appear to experience T3a up-staging after PN with increasing initial T stage. Up-staged pT3a patients have worse cancer specific survival after PN compared to those with similarly sized localized tumors. Furthermore, the up-staged pT3a patients after PN appear to experience similar survival to pT3a patients undergoing RN. However, pT3a patients undergoing PN had worse survival with increasing tumor size, reinforcing the need for improvements in preoperative staging and identifying patients at risk of up-staging.

